# The Challenge of Eight Years as *Arquivos Brasileiros de Cardiologia* Editor-in-Chief

**DOI:** 10.5935/abc.20170190

**Published:** 2017-12

**Authors:** Luiz Felipe P. Moreira

**Affiliations:** Instituto do Coração - Hospital das Clínicas HCFMUSP - Faculdade de Medicina - Universidade de São Paulo, São Paulo, SP - Brazil

**Keywords:** Cardiovascular Diseases, Cardiology, Periodicals as Topic

When we took over as Editor-in-Chief of *Arquivos Brasileiros de Cardiologia* in January 2010, the journal was more than 60 years old and had already attained complete maturity from the huge work developed by the great masters of the Brazilian cardiology who had preceded us as Editors. Present in the most relevant international citation indexing services, the *Arquivos Brasileiros de Cardiologia* is the major outreach channel of the Brazilian scientific research in cardiovascular diseases, representing the major asset of the Brazilian Society of Cardiology. Several suggestions were implanted aimed at consolidating our editorial structure and reaching the position as the major scientific forum in cardiology in Latin America.^1^

In the past eight years, the trajectory of the *Arquivos Brasileiros de Cardiologia* was characterized mainly by actions to improve its editorial process and to incorporate new edition technologies and new digital media. To face the great demand of the manuscripts submitted to publishing, which nowadays add up to 500 articles per year, the *Arquivos Brasileiros de Cardiologia* improved their electronic submission system and the review and editing processes, ensuring faster responses to the authors and better editorial quality publishing of the articles selected. Currently, the mean time of assessment and correction of the articles is around four months, while that time for Portuguese and English language publications is around four to five months. In addition, we provide the authors with the previous publishing of their articles in PubMed within less than eight months from their initial submission, guaranteeing the researchers the fast insertion of their work in the international literature. In parallel, we strived to incorporate the major editorial practices of the most prestigious international journals, such as the policies recommended by the International Committee of Medical Journal Editors (ICMJE) and the Association of the Editors of the European Society of Cardiology-affiliated journals, in addition to the inclusion of processes of language review, statistical review and antiplagiarism. Available in the electronic pdf format since 2011, the *Arquivos Brasileiros de Cardiologia* have counted since July 2012 with formats compatible with most tablets and smartphones, facilitating integral access to the journal in the major electronic media access systems.

**Figure 1:**
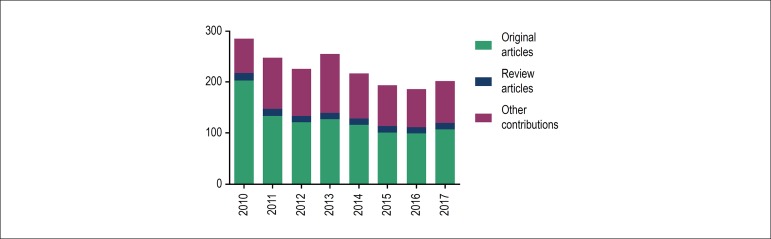
Number of articles published per year in the 2010-2017 period.

**Figure 2:**
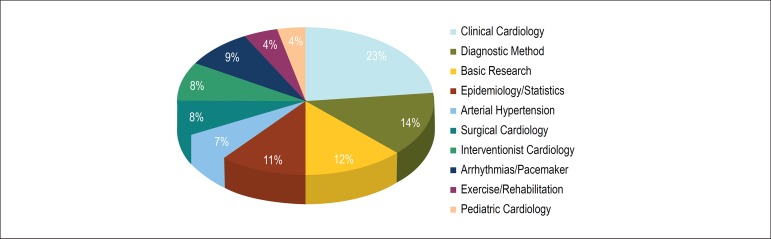
Percentage of articles published per area of insertion in the 2010-2017 period.

Over those eight years, the *Arquivos Brasileiros de Cardiologia* received more than 4700 scientific article submissions, being responsible for publishing 1813 articles between 2010 and 2017, of which, 1014 (56%) were contributions and 101 (6%) were review articles (Figure 1). Figure 2 shows the distribution of the articles published according to their area, under the responsibility of ten Associated Editors. The articles related to clinical cardiology, including the follow-up of patients with several illnesses, such as coronary artery disease and heart failure, represented 25% of the total number of articles published by the *Arquivos Brasileiros de Cardiologia*. Studies on basic research and diagnostic methods corresponded to more than 25% of the publications, while the other areas approached by the journal, such as epidemiology, hypertension, interventional cardiology, surgical cardiology, cardiac arrhythmias, pediatric cardiology and rehabilitation exercise, accounted for 4% to 11% of the manuscripts accepted. Those numbers represent one third of the articles submitted, with acceptance indices of 29% for original articles and 34% for review articles.

**Figure 3:**
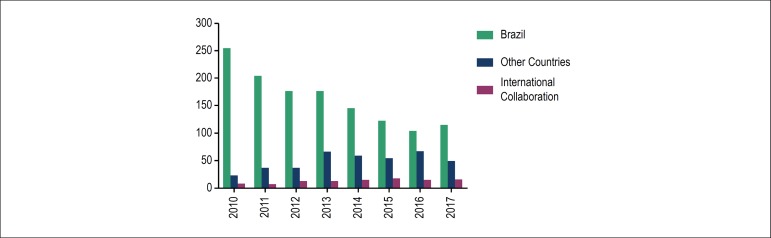
Number of articles published in the 2010-2017 period, according to the origin of the authors.

Approximately 80% of the articles published were submitted by Brazilian researchers, and more than 50% originated from postgraduation programs in Brazil. It is worth noting that, of the 3000 original articles published by Brazilian authors in journals indexed by the Thomson Reuters in the Web of Science database in cardiology and cardiovascular sciences between 2010 and 2017, 24% were published by the *Arquivos Brasileiros de Cardiologia*, evidencing the importance of that journal for the Brazilian cardiology.^2^ However, the number of articles submitted by researchers from other countries and studies developed in international collaboration has progressively increased over the years, written mainly by researchers from the United States, Portugal, Turkey, Spain, China and Canada (Figure 3). In addition, the significant elevation in the number of scientific articles currently submitted for publishing, resulting from the growing national and international scientific production, has progressively increased the rejection index of articles submitted by Brazilian researchers to the *Arquivos Brasileiros de Cardiologia*, hindering the publishing of dissertations and theses originating from Brazilian postgraduation programs in journals with international citation indexing.

**Figure 4:**
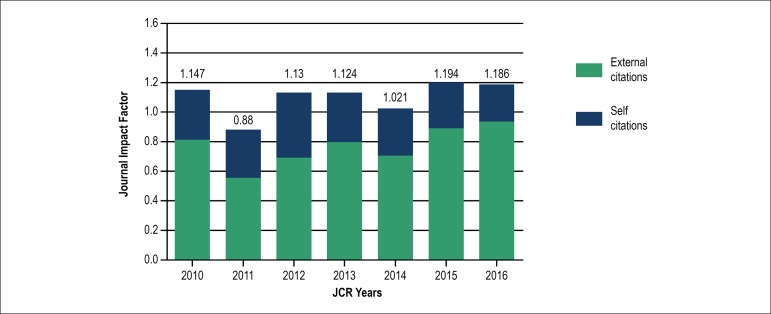
Evolution of the impact factor from 2010 to 2016, according to the “Journal of Citation Reports” of Thompson Reuters.

**Figure 5:**
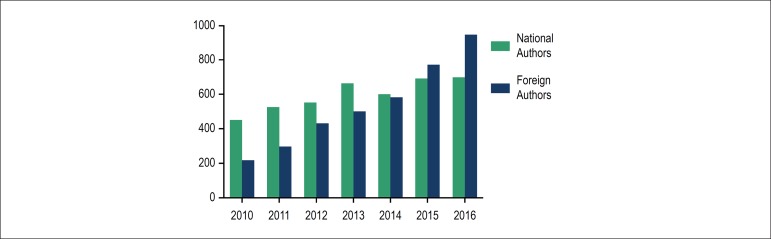
Number of citations obtained by the journal in the 2010-2016 period, according to the origin of the authors.

With the rise in the Brazilian and Latin-American scientific production on cardiology and cardiovascular sciences in the past decade, our rank in the international scenario improved, increasing the perspective of enhancing the qualification of our journals.^3^ However, the first impact factor of the *Arquivos Brasileiros de Cardiologia* was published in 2010, according to the database of the *Journal Citation Reports* of Thomson Reuters,^4^ and that factor remained stable over the past eight years, without a high citation rate of the journal itself (Figure 4). This occurred despite the progressive elevation in the number of citations attained by the articles published in *Arquivos Brasileiros de Cardiologia*, and it is worth noting that such elevation resulted from the increase in citations of foreign authors (Figure 5).

The lack of increase in the impact factor of the *Arquivos Brasileiros de Cardiologia*, similarly to what occurred with most journals published in Brazil, can be explained by the fact that the scientifically better qualified articles from Brazilian authors are mainly published in foreign journals, and that choice is justified by the higher impact factor of such journals.^5^ However, the citation indices attained by articles from Brazilian authors are lower than the international mean, even when published in journals of higher impact, indicating the need for higher appreciation of the journals published in Brazil, and this cannot represent a limitation for the dissemination of the articles published.^6,7^

However, it is worth considering the current existence of a large number of articles from Brazilian authors of significant scientific merit in cardiology, which is left with no appropriate outreach channel when rejected by the *Arquivos Brasileiros de Cardiologia*. With that in mind, four years ago, an increase in Brazilian cardiology publications under the responsibility of the Brazilian Society of Cardiology was proposed, with the formation of a family of journals, similarly to the major international journals. This initiative, which began with the transformation of the Department of Cardiovascular Image journal into the *ABC Imagem* journal, was not successful because of the submission of a reduced number of articles related to each specific area of knowledge, in addition to the difficulty of its indexing attributed to maintaining the journal’s name in Portuguese.

Contacting several Editors of international journals and the leaders of SciELO (*Scientific Electronic Library Online*), the major Latin-American platform for indexing and scientific journals, evidenced the need for a new journal with an English name and totally written in English, which is currently required for attaining indexing in the major international access systems. However, the scientific publications from countries with specific cultural characteristics, such as Portuguese-speaking countries, should be guided by policies that consider not only the need for wide content dissemination at international level but also the appropriate dissemination among their professional community members.^8^ We identified that our leaders and associates wanted to maintain the name of our major journal in Portuguese, going along with the preservation of its 70 years of history and the perspective to continue to have its content disseminated to all Brazilian cardiologists.

In the beginning of 2016, the Board of the *Arquivos Brasileiros de Cardiologia* (CONDARQ) defined the feasibility of a new journal with an English name and written in English, as well as the maintenance of the current characteristics of our major journal. This, following the guidance of the SciELO leaders, would open the perspective of rapid indexing of the new journal, which would remain affiliated to the *Arquivos Brasileiros de Cardiologia*, although having its own name, and there would be no competition between the associated journals, despite the similar content of the publications. This is ensured by the joint administration of both journals by the Brazilian Society of Cardiology, by the common policies of submission and peer review, and by the different levels of indexing and impact factors of the journals. In addition, the joint management of the journals reduces the financial costs of their maintenance, increases agility and sharing of the editorial and peer-review processes, magnifies their visibility due to the concomitant dissemination of their contents, in addition to encouraging the scientific debate related to the studies published.

Based on those arguments, the CONDARQ decided to incorporate the *International Journal of Cardiovascular Sciences*, then published by the Rio de Janeiro State Society of Cardiology, into the Brazilian Society of Cardiology. As predicted in the SciELO guidance, the incorporation of the new journal into the Brazilian Society of Cardiology facilitated its final indexing in that international platform, which happened less than one year from the beginning of the process mentioned, opening the perspective of its rapid indexing by the PubMed, PubMed Central and Scopus systems.^9^

During 2016, the bases for transferring the editorial process of the *International Journal of Cardiovascular Sciences* to the group responsible for the *Arquivos Brasileiros de Cardiologia* were defined, and both journals were integrated, with the construction of common access pages and submission systems. The priority of the *Arquivos Brasileiros de Cardiologia* regarding the manuscript submission system was defined, as were the mechanisms to direct at the *International Journal of Cardiovascular Sciences* those relevant manuscripts whose publishing was rejected by the major journal. This mechanism accounted for the referral of more than 150 articles to the new journal in the past 15 months, contributing to its regular publishing and to indexing a significant number of Brazilian articles, which otherwise would not have the perspective of rapid dissemination in indexed international journals, many of them originating from postgraduation programs. Finally, the future Editors-in-Chief of both journals were concomitantly approved through an exam-based selection process for the 2018-2021 period, and aspects regarding the interaction between the journals were considered during the process. The definitive implantation of that project, following the instructions of the major international indexing systems, awaits final approval by the Brazilian Society of Cardiology, leaving that important perspective still open.

Based on the trajectory of the *Arquivos Brasileiros de Cardiologia* in the past eight years, we claim that we hand a structured journal over to the Brazilian Society of Cardiology, in accordance with the editorial recommendations followed by the major international scientific journals, with adequate agility and infrastructure to keep its position as the major outreach channel of the Brazilian cardiology and cardiovascular science. However, we could neither effectively internationalize it, nor complete the restructuring process of the Brazilian Society of Cardiology publishing policies, which is undoubtedly fundamental for the scientific growth of cardiology and for the formation of new researchers through new postgraduation programs.

By concluding the challenging mission of conducting the *Arquivos Brasileiros de Cardiologia* during the 2010-2017 period, we leave an even greater challenge to the next Editor. The future expectations of that journal include the continuing growth of its internationalizing process, increasing the participation of the Associated Editors, reviewers and foreign authors. However, this should happen without putting aside the mission of the Brazilian Society of Cardiology scientific publishing, which continues to represent the major outreach channel of the Brazilian cardiology and cardiovascular sciences.
